# Outcomes of Recurrent Head and Neck Cutaneous Squamous Cell Carcinoma

**DOI:** 10.1155/2011/972497

**Published:** 2011-06-09

**Authors:** Nichole R. Dean, Larissa Sweeny, J. Scott Magnuson, William R. Carroll, Daniel Robinson, Renee A. Desmond, Eben L. Rosenthal

**Affiliations:** ^1^Division of Otolaryngology, Head and Neck Surgery, Department of Surgery, The University of Alabama at Birmingham, Volker Hall G082,1670 University Boulevard, Birmingham, AL 35233, USA; ^2^Division of Preventive Medicine, Department of Medicine, University of Alabama at Birmingham, Birmingham, AL 25294, USA

## Abstract

Recurrent, advanced stage cutaneous squamous cell carcinoma (cSCC) is uncommon with limited publications on patient outcomes. A retrospective study including patients who underwent surgical resection for recurrent, advanced stage cSCC of the head and neck was performed (*n* = 72). Data regarding tumor site, stage, treatment, parotid involvement, perineural invasion, positive margins, metastasis, and disease-free survival was analyzed. The majority of patients were male (85%) and presented with recurrent stage III (89%) cSCC. Two-year disease-free survival was 62% and decreased to 47% at 5 years. Parotid involvement, positive margins, nodal metastasis, or the presence of perineural invasion did not correlate with decreased survival (*P* > .05). Distant metastasis was a strong indicator of poor overall survival (*P* < .001). Adjuvant postoperative radiotherapy did not improve overall survival (*P* = .42). Overall survival was poor for patients with advanced recurrent cSCC despite the combined treatment with surgery and radiotherapy.

## 1. Introduction

Nonmelanoma skin cancer (NMSC) is the most common diagnosed malignancy in the United States with more than one million new cases reported each year [1]. Basal cell (BCC) and cutaneous squamous cell carcinoma (cSCC) comprise nearly all of the NMSCs. The majority of lesions (80–90%) arise in the sun exposed areas of the head and neck [2] and are successfully treated by complete tumor excision. A small percentage of NMSCs, mostly cSCCs, are refractory to standard surgical resection [1, 3]. Several outpatient-based studies have demonstrated a low incidence of nodal metastasis (2-3%) in patients with cSCC [4, 5]. Most tertiary care center-referred patients, however, present with recurrent disease and are at increased risk for neck metastasis, poor local control, and further cancer recurrence. High risk prognostic indicators include size, anatomic site, recurrence, history of radiation, immunosuppression, and perineural invasion [6]. It is unknown whether the same risk factors can be applied to determine prognosis in patients with recurrent disease.

In the present study, we evaluate outcomes in patients with advanced, recurrent cSCC of the head and neck. In the majority of cases, the treatment recommendations for this patient population are surgery and adjuvant radiotherapy [7]. Although no single variable can dictate treatment, this study sought to identify further predictive factors and provide guidance for counseling patients with aggressive head and neck cSCC.

## 2. Materials and Methods

A retrospective review of all patients (*n* = 72) who presented with recurrent, advanced stage (III or IV) cSCC of the head and neck between June 1998 and December 2007 was performed at The University of Alabama at Birmingham following Institutional Review Board approval. Tumors were staged according to the American Joint Committee on Cancer (AJCC) guidelines, and histology was confirmed by pathology. Tumors were divided into 5 different anatomic sites: face, neck, ear, periauricular area, and scalp. Lesions occurring on the face included forehead, periorbital, nose, lip, and chin. Periauricular lesions were defined as lesions occurring on the temple, cheek, or postauricular area. 

All patients underwent aggressive surgical resection. This included parotidectomy for cases in which parotid involvement was suspected on preoperative imaging or clinical exam. Parotidectomy ranged from superficial to radical parotidectomy with sacrifice of the facial nerve. The majority of patients required neck dissection and postoperative radiation. Selective, modified, or radical neck dissection was performed at the time of resection or in a staged procedure. Neck dissection was indicated when nodal metastasis was suspected on preoperative imaging or when the patient presented with an advanced T classification of their lesion. Postoperative radiation was recommended for patients with large cutaneous malignancies when more than one positive node was identified on neck dissection, when negative surgical margins could not be obtained, or in the presence of perineural or lymphovascular invasion. Histological margins were defined as negative if the advancing tumor edge was ≥4 mm from the line of surgical excision and positive if less than 4 mm. All cutaneous defects were repaired either by primary closure, split thickness skin graft, local or regional flap coverage, or free tissue transfer.

Demographic characteristics, including patient age, gender, a history of immunosuppression, previous treatment, and time to recurrence, were recorded. Prognostic indicators including tumor site, size, perineural invasion, positive margins, or histologic grade were reviewed. Outcomes measured consisted of disease-free survival and cancer recurrence. 

 Descriptive variables are reported as means (±SD) and categorical variables as percentages. Descriptive statistics were compared by general linear models for normally distributed variables or the Kruskal-Wallis test for otherwise. The relationship between patient clinical and treatment factors and disease-specific survival was calculated using the Kaplan-Meier method. Survival time was calculated as the interval from date of surgery to date of death or date of last followup. Deaths due to other causes were censored for these analyses. A *P*-value of <.05 was considered statistically significant. Statistical analysis was performed using SAS Version 9.2 software (SAS Institute Inc., Cary, NC).

## 3. Results

Between 1998 and 2007, there were 72 patients identified who underwent surgical resection for recurrent, advanced stage cSCC. The majority of patients were male (84.7%), presented with stage III disease (88.8%), and had undergone previous surgical resection (76.5%). Median time from previous skin cancer diagnosis to presentation with recurrence was 5.7 months (range, 1–41 months). Mean tumor size was 3.4 cm in largest dimension (±SD 2.07). The majority of lesions occurred on the ear or periauricular area (65.2%) and were classified as T4 lesions (83.3%) invading into deep extradermal structures (Table 1). Ten patients had a history of immune suppression due to lymphoma or leukemia (*n* = 6), rheumatoid arthritis (*n* = 1) or were currently maintained on immunosuppressive medications due to prior transplant (*n* = 3). 

No difference in margin status was observed between the various tumor sites (*P* = .48). Perineural invasion, a known risk factor for recurrence and metastasis, occurred in 36.9% of patients. Patients with cSCC of the ear and periauricular area were more likely to demonstrate perineural invasion on surgical pathology in comparison to all other sites (*P* = .06). Although perineural invasion did not significantly correlate with tumor site or size, patients with this finding were more likely to have parotid involvement (*P* = .04). A total of 39 patients (54.1%) underwent superficial (*n* = 16), total (*n* = 11) or radical (*n* = 12) parotidectomy. Four patients had undergone previous parotidectomy for positive nodal metastasis. Parotid involvement, either by direct extension or nodal metastasis, was confirmed by surgical pathology in 92.3% of cases (*n* = 36). 

The majority of patients with parotid involvement underwent neck dissection (92.3%). Neck dissection was performed in 66.7% of all patients. The majority (64.5%) underwent selective (*n* = 31) or modified radical (*n* = 14) neck dissection. One patient required a radical neck dissection. Another patient with a posterior scalp lesion had previously undergone an extended posterior triangle dissection. Three patients had undergone previous neck dissections. Positive nodal metastasis occurred in 43.7% (*n* = 21/48) of patients, and nearly all cases occurred in patients with advanced T classification (83.3%) and those with parotid involvement (85.7%, *n* = 18/21). The majority of nodes were located in levels I–III (95%). No positive nodes were identified in level IV, and one case of nodal metastasis occurred in level V (Table 3). Nodal metastasis did not correlate with tumor size, though a larger percentage of patients with cSCC of the ear demonstrated neck disease (66.7%) in comparison to other sites (*P* = .14). Eight patients had evidence of distant metastasis at the time of surgical resection. Distant metastasis did not correlate with perineural invasion, original tumor site, size, or parotid involvement at the time of surgical resection (*P* > .05). The histologic grades of the tumors consisted of 19% (*n* = 14) well differentiated, 56% (*n* = 40) moderately differentiated, 8% (*n* = 6) moderate-poorly differentiated, and 17% (*n* = 12) poorly differentiated. There was not a statistically significant relationship found between histologic grade and survival (data not shown).

All defects were closed either by primary closure or split thickness skin graft (*n* = 23), local or regional flap (*n* = 7), or free tissue transfer (*n* = 42). One patient underwent pectoralis major myocutaneous flap reconstruction, and 6 patients required local cervicofacial flap coverage. Patients with larger defects typically required free flap reconstruction. The radial forearm free flap (*n* = 13) and anterolateral thigh (*n* = 12) were most commonly utilized for soft tissue coverage. In cases in which bony reconstruction was also required, the osteocutaneous radial forearm free flap (*n* = 4) and fibula (*n* = 2) were employed (Table 2). 

The majority of patients underwent postoperative radiation (66.7%) as a result of advanced stage disease. Fifteen patients had undergone previous radiotherapy, and 3 underwent both pre- and postoperative radiation. The majority of patients with positive margins underwent postoperative radiation. Three patients had prior radiation therapy and were unable to tolerate the cytotoxic effects of a second course of radiation, and two electively declined further therapy. Three patients with perineural invasion did not undergo postoperative radiotherapy based on personal preference. 

Mean time to follow up was 18.5 months. Forty percent of patients (*n* = 28) developed local (67.8%, *n* = 19), regional (25%, *n* = 7) or distant metastasis (14.2%; *n* = 4) during the followup period. Median time to cancer recurrence was less than 7 months (6.5, range 1–41 months). One patient had local recurrence and developed lung metastasis while another patient developed both recurrent neck disease and lung metastasis. Although not statistically significant, patients were more likely to recur if they presented with cSCC of the ear or periauricular area (*P* = .06), demonstrated positive nodal metastasis at the time of neck dissection (*P* = .14), or did not undergo postoperative radiotherapy (*P* = .18). Margin status, a history of immunosuppression, and perineural invasion did not predict cancer recurrence. 

**Figure 1:**
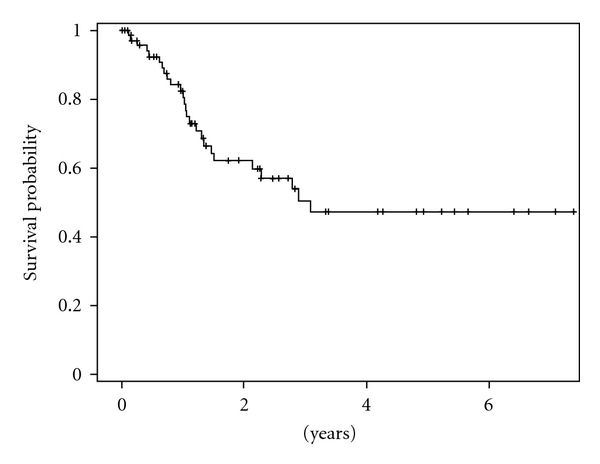
Overall disease-free survival for patients with advanced recurrent cutaneous squamous cell carcinoma.

**Figure 2:**
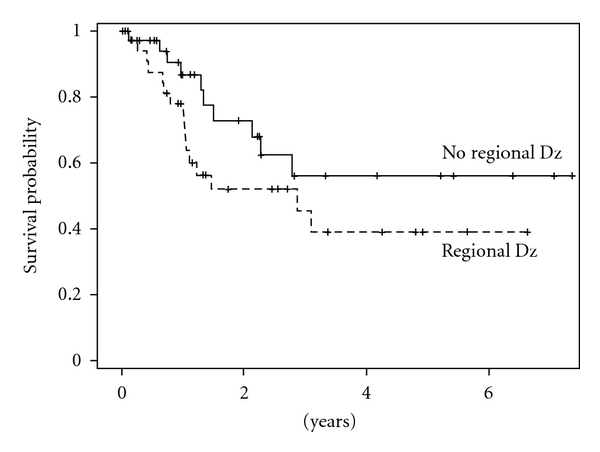
Disease-free survival for patients with and without positive nodal metastasis. Two-year disease-free survival for patients with regional disease was 47.7% versus 72.9% for those without regional metastasis (*P* = .14).

Two-year disease-free survival was 62.2% and was reduced to 47.2% at 5 years (Figure 1). Age greater than 65 (*P* = .34), male gender (*P* = .06), immunosuppression (*P* = .22), and patients with a previous history of radiation (*P* = .4) tended towards worse survival outcomes. Surprisingly, tumor characteristics including site, size (greater than 3.4 cm), parotid involvement, perineural invasion, or positive margins following resection had no influence on disease-free survival. Two-year disease-free survival for patients with positive nodal metastasis was 47.7% versus 72.9% for those without neck disease (*P* = .14) (Figure 2). Although locoregional metastasis was not a significant predictor of survival, distant metastasis noted within 30 days of surgical resection was associated with poor prognosis (*P* < .001). No patient with distant metastasis survived beyond 13 months. Most patients required free flap reconstruction and postoperative radiation for advanced disease. Patients who underwent free flap reconstruction tended towards worse survival outcomes (*P* = .24). Despite aggressive surgical resection, postoperative radiation was not shown to affect long term disease-free survival (*P* = .42) or repeat cancer recurrence (*P* = .85) for this patient population. Although not statistically significant, patients with cervical metastasis who underwent postoperative radiation had improved locoregional control (68% versus 25%, *P* = .14) when compared to those who underwent surgery alone.

## 4. Discussion

Nonmelanoma skin cancer is the most common malignancy worldwide with over 140,000 cases of cSCC diagnosed each year in the United States alone [8]. The incidence of regional metastasis among patients with cSCC ranges from 0.5 to 16% [4] and can result in potentially fatal consequences. Although a number of factors have been identified as high risk prognostic indicators for cancer recurrence and metastasis, most studies to date have only evaluated primary cSCC. In contrast, the majority of patients presenting to a tertiary care center have undergone previous skin cancer treatment and subsequently developed a second primary or recurrence. A diagnosis of recurrent cSCC alone confers a more aggressive tumor subtype [9], yet identifying factors associated with poor outcome is essential for patient management and decision making.

In the present study, we review tumor characteristics, prior treatment, and outcomes in patients with recurrent, advanced stage cSCC and evaluate the role of postoperative radiotherapy. Surgery combined with radiation is the recommended treatment in most cases of advanced disease [7]. In a study by Veness et al., improved locoregional control and disease-free survival (73% versus 54%, *P* = .004) were achieved in patients who received adjuvant radiotherapy compared to surgery alone [10]. In another series, 5-year disease-free survival was significantly improved for patients undergoing adjuvant radiotherapy following surgical resection (73% versus 18%, *P* = .001), and locoregional control was maintained in 77% of patients [11]. Both studies included patients with parotid or cervical metastasis. Nearly 50% of all patients in this study presented with parotid involvement, and 30% had cervical metastasis. All patients underwent surgical resection, and most (66.7%) had postoperative radiation. Postoperative radiation did not have an impact on overall disease-free survival (*P* = .42) or cancer recurrence (*P* = .85). Although not statistically significant, patients who presented with cervical metastasis and received adjuvant radiotherapy in addition to surgical resection had improved locoregional control when compared to those who underwent surgery alone (68% versus 25%, *P* = .14). Adjuvant radiotherapy appears to provide some benefit in patients with advanced, recurrent cSCC though the risk for repeat recurrence is high given the aggressive nature of these neoplasms. The role for systemic therapy in the treatment of recurrent, advanced stage cSCC remains unknown. Although there have been some case reports citing improved outcomes with the addition of cetuximab [12–14], the majority of publications on targeted therapies against EGFR and its tyrosine kinase have demonstrated limited improvement in the mortality of patients with advanced disease when used as monotherapy [15–19]. 

The median time from previous skin cancer diagnosis to presentation with initial recurrence was only 5.7 months (range, 1–41 months). This is likely due to the patient population being comprised of advanced, recurrent cutaneous squamous cell carcinomas, and as a result, this cohort of patients had very aggressive disease. The rapid recurrence of the disease reflects the malignant biology of late stage cSCC. In addition, nearly 40% of patients in this series developed a second recurrence with the majority occurring locally (68%). Distant metastasis noted in the immediate postoperative period was associated with poor prognosis (*P* < .001). Median time to cancer recurrence was 6.5 months, and overall 2-year disease-free survival was 62%. Although not statistically significant, patients from the present study were more likely to recur if they had cSCC of the ear or periauricular area (*P* = .06), demonstrated cervical metastasis on surgical pathology (*P* = .14), or did not undergo postoperative radiotherapy (*P* = .18). Previous studies have demonstrated a higher incidence of nodal metastasis among patients with lesions located on or around the ear as a result of lymphatic drainage to the parotid gland [20, 21]. In the present study, nearly half of all patients with parotid involvement either by direct extension or nodal metastasis also demonstrated cervical disease. Multiple studies have demonstrated that patients with parotid involvement are at a high risk for cervical metastasis [9, 22]. In a study by Ying et al., 44% of patients with parotid metastasis also had positive cervical nodes [23]. Therefore it is recommended that all patients with parotid metastasis undergo selective neck dissection. 

The majority of patients in this series underwent selective neck dissection which included levels I–III. Fifty percent of the nodal metastasis were located in level II. This finding is similar to what a recent article published, where nearly 80% of all positive nodal metastasis from cSCC were located in level II [24]. Although parotid involvement did not have an influence on disease-free survival in this patient population, the 2-year disease-free survival for patients with cervical metastasis was 48% versus 73% for those without neck disease (*P* = .14). In the study by Clayman et al., 3-year disease-free survival was 69% for patients with lymph node involvement versus 87% for those without nodal disease (*P* = .09) [25]. Cervical metastasis seemingly confers a worse prognosis for patients with recurrent disease. Appropriate neck dissection should be employed in patients with evidence of cervical metastasis or in the presence of parotid involvement. If parotid involvement occurs and a neck dissection is not performed then radiation should also be directed at the neck due to the high risk of cervical metastasis in patients with both primary and recurrent disease. 

Perineural invasion and tumor size have been considered high risk factors in patients with primary disease [9]. In a study by Clayman et al., patients with recurrent carcinoma were more likely to present with larger tumors which invaded beyond subcutaneous tissues and which demonstrated perineural (24%) and lymphovascular invasion [25]. Mean tumor size for patients presenting with recurrent disease in our study was 3.6 cm, and the majority were T4 lesions (83%). In addition, perineural invasion occurred in 37% of patients. Patients with cSCC of the ear and periauricular area were more likely to demonstrate perineural invasion on surgical pathology in comparison to all other sites (*P* = .06), and patients with perineural invasion were more likely to have parotid involvement (*P* = .04). It has previously been demonstrated that there is a significant increase in both nodal and distant metastasis for patients with perineural invasion when compared to those without [26]. Similarly, tumor size has been associated with an increased risk of metastasis: 30% for tumors >2 cm versus 9% for those <2 cm [27]. Perineural invasion and tumor size did not influence survival rates in this patient population. These results may be related to the fact that all these patients had high risk prognostic factors and received postoperative radiation which resulted in some improvement in locoregional control. It could also be that recurrent cSCC represents a more aggressive tumor subtype, and these patients all presented with advanced disease.

## 5. Conclusions

Recurrent advanced stage cSCC confers a poor prognosis with an increased risk for parotid involvement, nodal metastasis, and poor locoregional control. Despite aggressive surgical resection including parotidectomy and neck dissection followed by postoperative radiotherapy, 5-year disease-free survival rates are less than 50%. Patients presenting with recurrent disease should be appropriately counseled with regards to outcome and treatment recommendations.

##  Conflicts of Interest

The authors declare that there is no conflicts of interests.

## Figures and Tables

**Table 1:** Patient characteristics.

Characteristic	*n* (%)
*Age-years*	
Mean (range)	71 (42–93)
*Gender*	
Male	61 (85)
Female	11 (15)
*Tumor subsite*	
Face	19 (27)
Forehead	5
Periorbital	4
Nose	8
Chin	1
Lip	1
Periauricular	34 (47)
Cheek	13
Temple	18
Postauricular	3
Ear	13 (18)
Scalp	3 (4)
Anterior	2
Posterior	1
Neck	3 (4)
*T classification*	
T1	1 (1)
T2	5 (7)
T3	4 (6)
T4	60 (83)
Tx	2 (3)
*TMN Stage *	
III	64 (89)
IV	8 (11)

**Table 2:** Free flaps used for cutaneous defect reconstruction.

Flap type	*n*
ALT	12
Latissimus	3
Rectus	8
RFFF	13
Fibula	2
OCRFFF	4

ALT: anterolateral thigh; RFFF: radial forearm free flap; OCRFFF: osteocutaneous radial forearm free flap.

**Table 3:** Patterns of cervical lymph node metastasis.

Level	*n *(%)
I	6 (25)
II	12 (50)
III	5 (20)
IV	0 (0)
V	1 (5)
